# Updating of action–outcome associations is prevented by inactivation of the posterior pedunculopontine tegmental nucleus

**DOI:** 10.1016/j.nlm.2013.03.002

**Published:** 2013-05

**Authors:** Duncan A.A. MacLaren, David I.G. Wilson, Philip Winn

**Affiliations:** a Strathclyde Institute of Pharmacy & Biomedical Sciences, University of Strathclyde, 161 Cathedral Street, Glasgow G4 0RE, UK; b School of Psychology & Neuroscience, University of St Andrews, St Mary’s Quad, South Street, St Andrews, Fife KY16 9JP, UK

**Keywords:** Action–outcome, Learning, Pedunculopontine, Basal ganglia, Contingency degradation, Rat

## Abstract

The pedunculopontine tegmental nucleus (PPTg) is in a pivotal position between the basal ganglia and brainstem: it is able to influence and regulate all levels of basal ganglia and corticostriatal activity as well as being a key component of brainstem reticular and motor control circuitry. Consistent with its anatomical position, the PPTg has previously been shown to process rapid, salient sensory input, is a target for Parkinson’s disease treatments and has been implicated in associative learning. We explicitly investigated the role of the posterior pPPTg (pPPTg) in action–outcome processes, where actions are performed with the goal-directed aim of obtaining an anticipated outcome. We assessed rats’ sensitivity to degradation of the contingency between actions (lever pressing) and outcomes (food reward) during either inactivation of pPPTg by microinjection of the GABA agonist muscimol or control infusions of saline. In response to the degradation of contingency between lever press and food reward, saline treated rats rapidly reduced rates of lever pressing whereas muscimol treated rats (pPPTg inactivation) maintained previous lever pressing rates. In contrast, when the contingency between lever press and food reward was unchanged saline and muscimol treated rats maintained their previous rates of lever pressing. This shows that the pPPTg is critically required for updating associations between actions and outcomes, but not in the continued performance of previously learned associations. These results are consistent with a role for the PPTg in ‘higher-order’ associative learning and are the first to demonstrate a brainstem role in action–outcome learning.

## Highlights

• The pedunculopontine tegmental nucleus is essential for action–outcome learning.
• Sensitivity to instrumental contingency degradation is blocked by PPTg inactivation.
• Inactivation of PPTg does not change performance of previously learnt operant tasks.
• This is the first demonstration of a role for brainstem in action–outcome learning.
• Learning functions of basal ganglia extend into the deepest parts of the circuitry.

## 1 Introduction

Understanding brain mechanism of action–outcome (A–O) learning is of both theoretical and practical importance: learning the causal relationship between actions and outcomes is essential for adaptation and survival, and dysfunction of associative relationships features in diseases including schizophrenia and addiction (Everitt & Robbins, 2005; Murray, Corlett, & Fletcher, 2010). Several structures have been implicated in action–outcome learning: the posterior dorsomedial striatum (Yin, Ostlund, Knowlton, & Balleine, 2005), prelimbic cortex (Corbit & Balleine, 2003), mediodorsal thalamus (Corbit, Muir, & Balleine, 2003) and entorhinal cortex (Corbit, Ostlund, & Balleine, 2002; Lex & Hauber, 2010). These are all part of an extended corticostriatal circuitry critical for developing responses to new stimuli as well as maintaining habitual responding and selecting actions where there are multiple choices (Everitt & Robbins, 2005; Redgrave, Vautrelle, & Reynolds, 2011).

We investigated the hypothesis that the posterior pedunculopontine tegmental nucleus (pPPTg) contributes to A–O learning. There are several reasons for believing that it may: (i) Anatomically, pPPTg can be considered a functional component of the basal ganglia family (Mena-Segovia, Bolam, & Magill, 2004) with ascending connections to midbrain dopamine (DA) neurons and thalamus (Maskos, 2008) and reciprocal connections with globus pallidus, subthalamus and extended amygdala (Winn, 2006; Zahm, Williams, Latimer, & Winn, 2001). This means the pPPTg has access to the extended corticostriatal circuitry previously implicated in A–O learning, as described above. (ii) Single-neuron recording studies in primate show that subpopulations of PPTg neurons selectively respond to cues that predict reward or to the actual delivery of reward (Okada, Toyama, Inoue, Isa, & Kobayashi, 2009) indicating that the PPTg can recognize the significance of reward-related sensory events. (iii) Rats with excitotoxic lesions of the PPTg are impaired at learning to lever press for intravenous amphetamine, but show no impairment if they have learnt a lever-reward association prior to lesion (Alderson, Latimer, Blaha, Phillips, & Winn, 2004). Furthermore, rats with lesions restricted to the pPPTg (which receives fast sensory information and projects to VTA and SNc (Winn, 2006)) also show impaired learning: rats are slow to learn to lever press for reward and slow to alter behavior in response to changes in reinforcement schedules (Wilson, MacLaren, & Winn, 2009a). While these studies show impairment in reinforcement behavior, they have not explicitly manipulated A–O learning.

A defining feature of intact A–O learning is sensitivity to changes in the contingency between performing the action and receiving the outcome. If the contingency is degraded (for example by delivery of outcomes not contingent on action) behavior is modified and the previously required action reduced. We sought to investigate the role of the pPPTg in A–O learning using a modified version of an established contingency degradation paradigm (Yin et al., 2005). We hypothesized that rats with pPPTg inactivation (created by microinjection of the GABA agonist muscimol) would be insensitive to degradation in contingency, demonstrating impaired updating of A–O associations.

## 2 Materials and methods

### 2.1 Subjects

Forty male Lister Hooded rats (Harlan Olac Ltd., Bicester, UK: mean surgery weight 404 g) were single housed in a temperature and humidity controlled room. Lights were on a 12-h light/dark cycle; testing was in the light phase. Prior to surgery and during recovery rats had free access to food and water. Four days prior to behavioral testing food was restricted to 15 g/rat/day standard lab chow, 7 days/week. Throughout, bodyweight was monitored daily to ensure it did not fall to below 85% of free-food weight. These experiments complied with the UK Animals (Scientific Procedures) Act 1986 and European Communities Council Directive of 24/11/86 (86/609/EEC).

### 2.2 Surgery

Rats were anaesthetized (Isoflurane; Abbot Laboratories, Maidenhead, UK) in an induction box before being placed in a stereotaxic frame (David Kopf, Tujunga CA, USA) and adjusted such that they were in the flat skull position. Pre-surgical analgesic (Rimadyl; 0.05 ml/rat; 5% w/v carprofen; Pfizer, Kent, UK) was injected subcutaneously. Craniotomies were drilled over the stereotaxic co-ordinate for pPPTg (+0.4 mm from interaural line; ±1.9 mm from midline) and 6 stainless steel mounting screws (Plastics One, Roanoke, VA, USA) fixed onto the skull. Bilateral guide cannulae (22 ga, 3.8 mm apart, protruding 5.0 mm below skull surface; Plastics One) were positioned using a stereotaxically mounted holder (Plastics One) and fixed onto the skull and screws using dental acrylic (Simplex Rapid; Kemdent Works, Wiltshire UK). Internal dummy cannula (protruding 1.0 mm from the guide cannulae; Plastics One) were inserted into the implanted cannulae and a cap (Plastics One) screwed on top. After being removed from the stereotaxic frame rats were given Hartmann’s solution to aid recovery (1 mL, i.p. Baxter Healthcare, Norfolk, UK). Every 2 days, dummy cannulae were changed with clean replacements. Rats were given at least 7 days recovery from surgery prior to operant training.

### 2.3 Operant training

Operant procedures were carried out in test chambers situated inside light and sound attenuating boxes (Med-Associates, St. Albans, Vermont, USA). In each box there were 2 retractable levers either side of a reward magazine. A houselight was located at the top of the opposing wall. No other light or sound generating devices were used. The contingency degradation behavioral protocol was a modification of that used by Yin and colleagues (Yin et al., 2005): rather than using 2 levers giving either a liquid or dry reward and then degrading the contingency of one lever, we had one lever deliver the same dry reward then in particular groups of rats degraded the contingency between lever press and outcome. This modification was based on pilot studies which established that with our standard operant box delivery mechanisms rats develop a strong preference for one reward type – liquid or dry pellet – which overrides the attempt selectively to manipulate the value or contingency of the rewards. Two days prior to training, each rat received two exposures to 1 g of testing pellets (Test Diet purified rodent tablet 5TUL, Sandown Scientific, Middlesex, UK) in their homecage; on the following day rats were placed in the operant boxes where 25 pellets were freely available in the reward magazine and no levers were extended. This allowed rats to become familiarized to the operant box, location of reward delivery and reduce neophobia to the pellets. Daily training sessions (40 min duration) began the following day, starting with a fixed-ratio 1 (FR1) schedule: 1 lever press on the active lever always produced 1 food pellet. Pressing on the inactive lever (side counterbalanced across rats) was recorded but had no consequence. Once rats met the performance criteria on FR1 (2 sessions >70 rewards) they were advanced onto a random-ratio 5 schedule (RR5; a 1:5 probability that a single pellet would be delivered per lever press) (criteria: 2 sessions >60 rewards), then onto RR10 (criteria: 2 sessions >20 rewards) and then RR20 schedules (2 sessions >20 rewards).

### 2.4 Contingency degradation training

Once rats met criteria on RR20 they were randomly assigned to either a saline or muscimol group and then further assigned to a contingent or non-contingent subgroup. The contingent groups were subsequently trained in a regime where pellet delivery was still dependent on lever pressing on RR20 (that is, no change in contingency from previous training). The non-contingent groups were trained in a regime where, every second, pellets were delivered with equal probability (1:34, determined by pilot studies to deliver approximately the same number of pellets to rats on the non-contingent schedule as that earned by rats on the RR20 schedule) whether the rat responded appropriately or not. Training sessions lasted 20 min and started 15 min after saline/muscimol infusion. Infusions were made via bilateral cannulae (3.8 mm apart, protruding 7.5 mm from the base; Plastics One) that were inserted into the implanted guide cannulae while the rats were lightly restrained. Injectors were attached by polyethylene tubing (PE50; Plastics One) to 2 syringes (1 μL, 23 ga needle, SGE Analytical Science, Victoria, Australia) driven by a syringe pump (PHD 2000, Harvard Apparatus, Holliston, MA, USA). Infusions of 0.3 μL of muscimol (0.05 μg in 0.3 μL saline; Tocris Bioscience, Bristol, UK) or saline (0.3 μL; Baxter Healthcare Ltd.) were made over 1 min and injectors were left in place for 1 min post-infusion before being removed and replaced with dummy cannulae. Training during these sessions was conducted every other day to allow for drug clearance.

### 2.5 Extinction test

After the third contingency training session rats were tested, without infusion, in a 20 min extinction test. In the same manner as in all testing sessions, both levers were extended and the houselight illuminated, but no rewards were delivered.

### 2.6 Histology

At the end of behavioral testing, rats were given a lethal i.p. injection of Euthatal (0.7 mL per rat; 200 mg/mL sodium pentobarbitone; Merial Animal Health Ltd., Harlow, UK) and transcardially perfused with phosphate buffered saline followed by fixative (4% paraformaldehyde in 0.1 M phosphate buffer). Brains were stored in sucrose solution (20% in 0.1 M phosphate buffer) before being cut on a freezing microtome. Coronal 30 μm sections were taken from the anterior cerebellum through to posterior substantia nigra. Parallel sections (1:4) were immunohistochemically processed to stain for choline acetyltransferase (ChAT), using goat anti-ChAT polyclonal antibody (Chemicon International Inc., Temecula, CA, USA), a Vector Labs “Elite” ABC kit (Peterborough, UK) and Sigma Fast DAB peroxidase substrate, before being mounted onto glass slides. Slides were examined under a light microscope (Leica DM LB2). Cannulae tip location was determined by examining evidence of injector track marks. The pPPTg contains a homogenous population of neurons – cholinergic, glutamatergic and GABA-ergic (among others). The cholinergic neurons are densely packed in pPPTg so its location was judged with reference to these, visible on the ChAT immunostaining. All cannulae within the area covered by these neurons were considered to be appropriately located.

### 2.7 Behavioral data analysis

Statistical analysis was performed in PASW 18.0 (SPSS Inc., Chicago, Illinois USA). Repeated measures ANOVA were performed across the degradation sessions. Univariate ANOVAs were performed to compare pressing rates between saline and muscimol contingent and non-contingent groups on the final day of pre-training and during extinction testing. In cases of significant group differences or interactions, these were investigated with univariate ANOVAs, protected planned pairwise comparisons and Bonferroni corrected *t*-tests, where appropriate. All effects were considered statistically significant when *p* ⩽ 0.05.

## 3 Results

### 3.1 Histological results

**Fig. 1:**
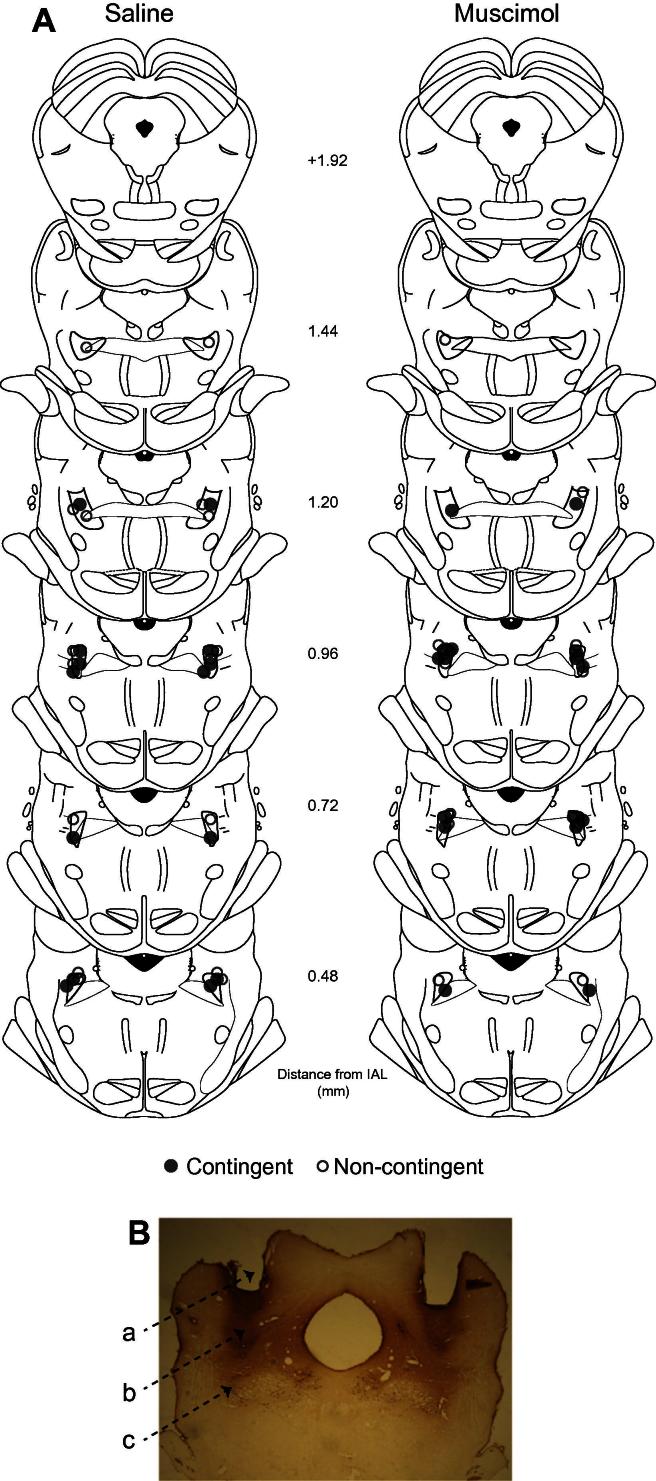
Cannulae placements. (A) Location of injector tips, shown on coronal sections adapted from the stereotaxic atlas of Paxinos & Watson (2005). The location of each tip is represented by a closed circle (contingent group) or an open circle (non-contingent group). The PPTg is outlined in dark gray; numbers indicate distance from interaural line (mm). (B) Photograph of representative ChAT immuno-stained section showing (a) the location of the guide cannula, (b) the dummy cannula and (c) the injector track ending in the ChAT+ neurons of the pPPTg.

Twenty-nine rats had cannulae tips located within the pPPTg (Fig. 1) giving final group sizes of: saline *n* = 14 (contingent *n* = 7; non-contingent *n* = 7); muscimol *n* = 15 (contingent *n* = 8; non-contingent *n* = 7). All rats had similar levels of guide, dummy and injector track damage, as illustrated in Fig. 1. The remaining rats were excluded from all analysis due to: complications with infusions (*n* = 4); tissue damage causing lesion (*n* = 4); cannulae missing pPPTg (*n* = 2) or not reaching a stable performance on RR20 (*n* = 1).

### 3.2 Behavioral results

#### 3.2.1 Training

Rats reached criterion on the RR20 training schedule after a mean of 11 (S.E. ± 0.27) sessions. Multivariate ANOVA confirmed there were no group differences pre-infusion (data not shown).

#### 3.2.2 Contingency training

**Fig. 2:**
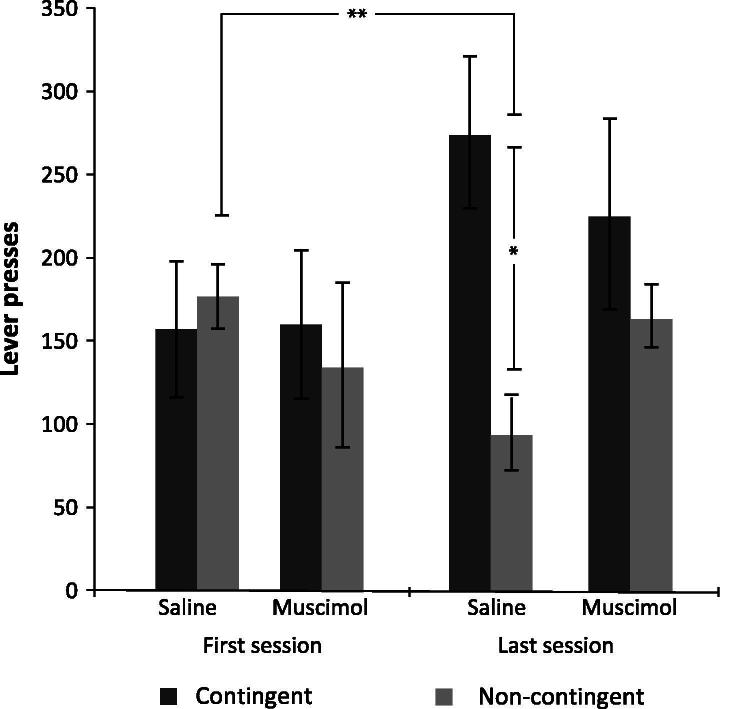
Contingency degradation sessions. Mean number of lever presses on the active lever for each group during the first and last of the three contingency degradation training sessions. * indicates *p* < 0.05, ** indicates *p* < 0.01. Error bars show S.E.M.

The effects of intra-pPPTg muscimol and saline on lever pressing in the contingency degradation sessions are shown in Fig. 2. Repeated measures ANOVA across the 3 degradation training sessions found no overall effect of infusion group, but a significant interaction between infusion group × contingency group × session (*F*_2,50_ = 3.56; *p* = 0.037). Post-hoc paired samples *t*-tests were performed comparing rates of lever pressing on first and last sessions. Pressing in the saline non-contingent group was significantly less during session 3 compared to 1 (*t*_6_ = 5.39; *p* = 0.008), but no other groups had a significant change in rate of lever pressing. Control rats, therefore, were able to learn the changing contingency between lever press and outcome whereas rats with inactivated pPPTg were unable to learn these changed contingencies. Similarly, univariate ANOVA of the last session found a main effect of group (*F*_3,25_ = 3.53, *p* = 0.029) and restricted pairwise comparisons revealed that the difference between saline contingent and non-contingent lever pressing was significant (*p* = 0.005) whereas the difference between the muscimol contingent and non-contingent pressing was not (*p* = 0.294). Further, the difference between saline and muscimol contingent pressing was also not significant (*p* = 0.399). Together, these results show that inactivation of pPPTg did not affect lever pressing ability per se, but selectively blocked the change in rates or lever pressing in response to the change in contingency between lever press and reward delivery. Throughout the degradation sessions all groups had low levels of inactive lever pressing, with muscimol groups having numerically higher (but not significantly higher) levels than saline groups. Results are summarized in Table 1.

#### 3.2.3 Extinction test

**Fig. 3:**
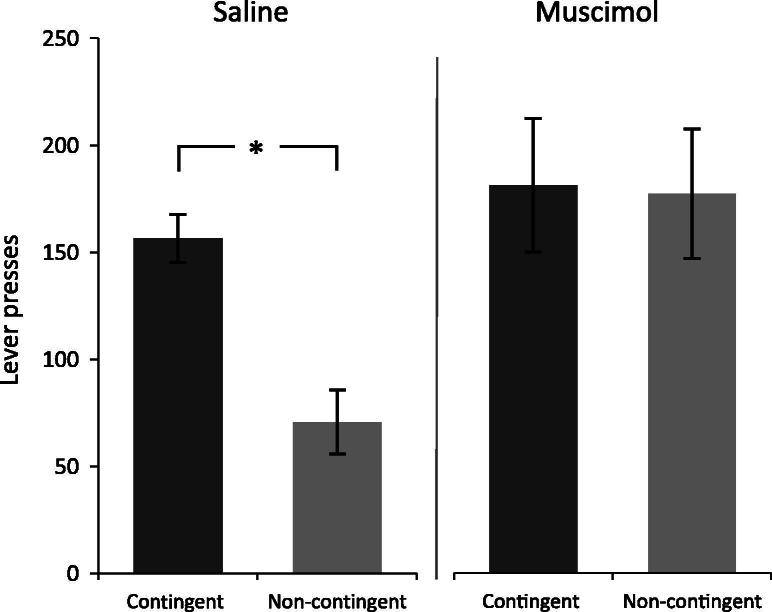
Extinction session. Mean number of lever presses on the active lever for each group during the extinction test. * indicates *p* < 0.05 Error bars shown S.E.M.

Performance during the extinction test followed the same pattern as that seen on the last day of contingency training (Fig. 3). Univariate ANOVA showed an infusion group × contingency interaction (*F*_3,25_ = 3.01, *p* = 0.012); restricted pairwise comparisons confirmed that saline treated rats pressed significantly less in the non-contingent group compared to the contingent group (*p* = 0.018) whereas there was no difference between rates of pressing in the contingent and non-continent muscimol treated groups (*p* = 0.906). The difference between saline contingent and muscimol contingent pressing was also not significant (*p* = 0.488).

### 3.3 Results summary

These results show a clear pattern: inactivation of the PPTg blocks sensitivity to contingency degradation during both degradation training and the extinction test, while having no effect on the continued performance of previously learnt contingent lever pressing.

## 4 Discussion

Using a contingency degradation paradigm, we have shown that a functioning pPPTg is critical for updating A–O associations – without the pPPTg this operation does not take place. In this paradigm the relationship between the action (lever pressing) and outcome (pellet delivery) was degraded such that, in the degraded condition, pellets were delivered at the same frequency whether the rats responded correctly or not. Rats with intact monitoring of their actions and their associated outcomes are sensitive to this change in contingency and consequently reduce the number of actions they perform. This pattern was found in saline infused control rats: non-contingent lever pressing was significantly lower than contingent after degradation and during the extinction test. In contrast, pPPTg inactivation by muscimol blocked sensitivity to contingency degradation since lever pressing was not significantly different between the contingent and non-contingent groups at any point. Importantly, this was not reflective of a deficit in the ability to lever press because rats in the muscimol-contingent group pressed at the same rate as rats in the saline-contingent group. Rather, it was a deficit in adapting behavior in response to the change in contingency, a defining characteristic of impairment in updating the association between actions and outcomes. This finding establishes PPTg as the first brainstem structure critically involved in A–O learning. Furthermore, it is the only identified structure outside classical corticostriatal circuitry involved in A–O learning that is able also to regulate the activity of this circuitry, including connecting midbrain DA neurons.

These results extend significantly previous studies showing learning impairments after PPTg lesions. Keating and Winn (2002) showed that despite normal movement rats with bilateral excitotoxic PPTg lesions were unable to learn or perform radial maze tasks in which reward location varied on every trial, making the relationship between action (which arm of the maze to enter) and outcome (successful reward retrieval) unpredictable. Alderson and colleagues (Alderson et al., 2004) found that rats with PPTg lesions were impaired at learning to lever press on FR2 for intravenous amphetamine (although unimpaired if they had learned prior to lesion that lever pressing was rewarded). However, both naïve and trained PPTg lesioned rats were unable to respond properly when on a progressive ratio schedule of reinforcement in which the relationship between outcomes and actions (the number of presses required) constantly changed. More recently, we have shown that pPPTg (and not anterior PPTg) lesioned rats were slow to learn to lever press during the initial stages of simple operant learning, and though with extended training they did learn the task, they were subsequently slow to adapt their rates of lever pressing in response to changes in reinforcement schedule (Wilson et al., 2009a). These findings are now potentially explainable as a deficit in A–O learning, since the updating of A–O associations is exactly what is required during the initial learning of new reinforcement schedules. Another requirement for normal learning and performance of operant tasks is the intact representation of the incentive value of the outcome and motivation to work for it. That rats in both the saline and muscimol contingent groups continued to lever press at the same rate – and therefore worked as hard for the same reward – shows that pPPTg inactivation does not affect motivation to work for reward.

The pPPTg is well positioned to contribute to formation of A–O associations because it has strong cholinergic and non-cholinergic projections to VTA and SNc DA neurons (Charara, Smith, & Parent, 1996; Oakman, Faris, Kerr, Cozzari, & Hartman, 1995). Phasic activity of these DA neurons in response to reward related sensory input leads to adaptation of firing patterns during learning about the events leading up to reward acquisition – initial phasic firing in response to unpredicted reward declines as phasic firing in response to stimuli that predict the reward develops (Schultz, 2002). Absence of the expected reward after a reward-predicting stimulus leads to decreased firing at the time of expected reward delivery. This phenomenon – the reward prediction error (RPE) signal – is considered to form the basis of reward related learning (Schultz & Dickinson, 2000) and target projection areas in the dorsal medial striatum have been shown to be critical for A–O learning (Yin et al., 2005). Midbrain RPE signal responses are fast (70–100 ms), leading to speculation about the source of information used for their calculation. Striatal, subthalamic, amygdala and cortical responses occur at similar or longer stimulus durations, making them implausible sources of functional input into VTA for RPE calculation (Redgrave, Gurney, & Reynolds, 2008). Other short latency sources of input to midbrain DA – for example the superior colliculus – are considered to contain insufficient information for calculation of RPE (Redgrave et al., 2008). However, primate electrophysiological experiments show that during a reward-driven behavioral task, different populations of PPTg neurons fire in response to stimuli predicting reward or to actual reward delivery (Okada, Nakamura, & Kobayashi, 2011; Okada et al., 2009). Furthermore, firing rate was dependent on reward magnitude – stimuli predicting large reward led to greater firing than stimuli predicting small reward. While there was no evidence of reduction or habituation of PPTg responses to continued presentation of the same stimulus, PPTg responses rapidly adapted to reversal of stimulus-reward pairings. PPTg, therefore, appears to contain information necessary for calculation of the RPE signal and has dynamic updating of firing patterns in response to changes in stimulus-reward (Okada et al., 2011) and context-reward (Norton, Jo, Clark, Taylor, & Mizumori, 2011) associations. In the rat it has been shown that PPTg neurons fire to reward-related sensory events before midbrain DA neurons do, and inactivation of PPTg suppresses the learned sensory responses of midbrain DA neurons to reward predicting stimuli (Pan & Hyland, 2005); this is consistent with information flow from PPTg to midbrain DA neurons. The current finding that inactivation of the pPPTg blocks updating of A–O associations is consistent with what would be predicted if the VTA was no longer receiving the necessary information for RPE calculation.

Another possible interpretation is that the saline non-contingent rats perceived the change in contingency as an indicator that they were now in a different context where rewards came freely without the requirement of lever pressing. PPTg has been shown to process reward related information in a context dependent manner (Norton et al., 2011), therefore it is worth considering whether rats with pPPTg inactivation continued lever pressing due to blocking of this contextual processing. However, the only prompt to the saline-non-contingent rats that the context had changed would have been be the change in contingency between action and outcome (all other operant testing conditions were identical). Therefore, whether directly through blocking action–outcome associations, or through blocking context-action–outcome associations, the result of pPPTg inactivation is still inability to update the casual relationship between the requirements of performing actions in order to obtain outcomes. It is also worth noting that PPTg lesioned rats form normal conditioned place preference for sucrose solution and cocaine (Alderson, Jenkins, Kozak, Latimer, & Winn, 2001; Parker & van der Kooy, 1995) suggesting normal contextual processing is possible in the absence of a functioning PPTg.

The anatomical location of PPTg, and its connections with pontine reticular formation and other motor output sites of brainstem and spinal cord, together with rapid access to multimodal sensory information, make it a key brainstem structure. Moreover, the intricate reciprocal connections between PPTg, basal ganglia structures, thalamus and the corticostriatal and corticothalamic loops have led to the conclusion that PPTg should be considered a functional part of these (Mena-Segovia et al., 2004). The PPTg is uniquely placed in circuitry that processes sensory events and selects one out of many competing actions to perform. It contains the information required to perform independently a rapid ‘low level’ analysis of sensory events and trigger the immediate appropriate response, or alternatively filter and interface specific aspects of sensory information into basal ganglia and thalamocortical systems for more advanced processing (Wilson, MacLaren, & Winn, 2009b; Winn, Wilson, & Redgrave, 2010, chap. 23). This ability of PPTg to operate as part of a sensory gating mechanism has led to speculation about a relationship with the symptoms of schizophrenia: PPTg is involved in pre-pulse inhibition (Swerdlow & Geyer, 1993) and the auditory P50 (P13 in the rat) (Miyazato, Skinner, & Garcia-Rill, 1999) both of which have been considered as endophenotypes for the disease (Turetsky et al., 2007). Impairment in A–O updating can be construed as a deficit in monitoring actions, also impaired in schizophrenia (Turetsky et al., 2007). Taking into consideration that PPTg neurons have a role in the regulation of DA systems, one can speculate that aberrant functioning of the PPTg may lead to some of the cardinal sensory processing deficits seen in schizophrenia.

## Figures and Tables

**Table 1:** Summary of main results, “–” indicates no significant difference found.

	Change during degradation training	Performance on last day of degradation	Performance in the extinction test
*Saline*			
Contingent	–	Significant difference (*p* = 0.005)	Significant difference (*p* = 0.018)
Non-contingent	Significant reduction (*p* = 0.008)		

*Muscimol*			
Contingent	–	–	–
Non-contingent	–		
